# TRAIL and curcumin codelivery nanoparticles enhance TRAIL-induced apoptosis through upregulation of death receptors

**DOI:** 10.1080/10717544.2017.1384863

**Published:** 2017-10-10

**Authors:** Xi Yang, Zhaojun Li, Qinjie Wu, Shouchun Chen, Cheng Yi, Changyang Gong

**Affiliations:** a Department of Medical Oncology, Cancer Center, and State Key Laboratory of Biotherapy, West China Hospital, Sichuan University, Chengdu, China;; b Department of Radiotherapy, Hainan General Hospital, Haikou, China;; c Chengdu Huachuang Biotechnology Co. Ltd, Chengdu, China

**Keywords:** TRAIL, curcumin, nanoparticles, codelivery, death receptors, apoptosis

## Abstract

Active targeting nanoparticles were developed to simultaneously codeliver tumor necrosis factor-related apoptosis-inducing ligand (TRAIL) and Curcumin (Cur). In the nanoparticles (TRAIL-Cur-NPs), TRAIL was used as both active targeting ligand and therapeutic agent, and Cur could upregulate death receptors (DR4 and DR5) to increase the apoptosis-inducing effects of TRAIL. Compared with corresponding free drugs, TRAIL-Cur-NPs group showed enhanced cellular uptake, cytotoxicity and apoptosis induction effect on HCT116 colon cancer cells. In addition, *in vivo* anticancer studies suggested that TRAIL-Cur-NPs had superior therapeutic effect on tumors without obvious toxicity, which was mainly due to the high tumor targeting and synergistic effect of TRAIL and Cur. The synergistic mechanism of improved antitumor efficacy was proved to be upregulation of DR4 and DR5 in tumor cells induced by Cur. Thus, the prepared codelivery nanoparticles may have potential applications in colorectal cancer therapy.

## 1. Introduction

Colorectal cancer is the third most common cancer and a leading cause of cancer associated death in the world (Chen et al., 2016; Siegel et al., 2017). Although new strategies have been developed, chemotherapy still has drawn more attention in the treatment of colorectal carcinoma. However, due to the absence of tumor-targeting and nonspecific biodistribution, conventional chemotherapeutic drugs always present lower efficacy and undesirable side effects such as nausea and vomiting, diarrhea, bone marrow suppression, fatigue and neurotoxicity (Egan et al., 2004; Lammers et al., 2012). Tumor necrosis factor-related apoptosis-inducing ligand (TRAIL/Apo2L), known as a cytokine of the tumor necrosis factor (TNF) superfamily, is a safe anticancer agent, which can induce extrinsic pathway of apoptosis by interacting with the death receptor 4 (DR4) and death receptor 5 (DR5) in a broad spectrum of human malignancies without affecting most normal cells (Ashkenazi et al., 1999, 2008). Hence, TRAIL has been used in numerous human clinical trials (Ashkenazi, 2002). Unfortunately, the application of TRAIL in clinic has been hampered due to its short half-life and TRAIL resistance (Herbst et al., 2010; De Miguel et al., 2016b). More importantly, the monotherapy of TRAIL could not obtain optimal efficacy in most cases (Munshi et al., 2002; Singh et al., 2003).

Combination therapy is one of the most frequently used strategies to overcome above-mentioned obstacles, which has been proved to be more effective than monotherapy by achieving synergistic effects and reducing toxicity. TRAIL was found to have synergistic interaction with other drugs like Curcumin (Cur) (Jung et al., 2005, 2006; Yang et al., 2012; Ahn et al., 2014; An et al., 2014). Cur is a natural yellow pigment purified from the rhizome of the plant *Curcuma longa*, which has exhibited a wide range of pharmacological activities like antimicrobial, anti-inflammatory and anticancer without obvious side effects (Anand et al., 2008; Naksuriya et al., 2014). Several studies showed that Cur could sensitize TRAIL-mediated apoptosis via the upregulation of DR5 in different cancer cells (Jung et al., 2005; Kamat et al., 2009). Nonetheless, *in vivo* combinational administration of TRAIL and Cur remains a challenge owing to its poor bioavailability, extremely low aqueous solubility and rapid metabolism of Cur. In addition, there is no pledge that cancer cells will simultaneously receive optimal levels of each therapeutic drug with different pharmacokinetic and pharmacodynamics properties. Therefore, it is important to construct a codelivery system for successful combination therapy, which can offer higher selectivity, longer drug circulation time and the synchronous exposure of each drug (Peer et al., 2007; Torchilin, 2011).

Herein, we designed a codelivery nanoparticles system (TRAIL-Cur-NPs) based on biodegradable poly (ε-caprolactone)-poly (ethylene glycol)-poly (ε-caprolactone) (PCEC) triblock copolymer, which was loaded with soluble extracellular domain of TRAIL and Cur in nanoparticles. Poly (ε-caprolactone) (PCL) was utilized as hydrophobic segment, and poly (ethylene glycol) (PEG) was designed as hydrophilic segment to avoid the aggregation and clearance from the reticular-endothelial system (Photos et al., 2003; Shi et al., 2014). To best of our knowledge, this is the first reported TRAIL and Cur codelivery nanoparticles. Cur-loaded nanoparticles were formulated by modified solvent emulsion evaporation method, while TRAIL was bound on the obtained anionic Cur-loaded nanoparticles via electrostatic interactions. The ‘two-in-one’ strategy used to load Cur and TRAIL in TRAIL-Cur-NPs could efficiently improve the therapeutic effect of the drugs by prolonging their circulation time, enhancing the water dispersion of Cur and improving the active and passive tumor targeting. In TRAIL-Cur-NPs, TRAIL was used as both active targeting ligand and therapeutic agent, and Cur could upregulate death receptors to enhance the therapeutic effect. The findings of *in vitro* and *in vivo* anticancer studies using human colon cancer HCT116 cells confirmed that TRAIL-Cur-NPs showed an enhanced antitumor ability because of high tumor targeting and synergistic effect of TRAIL and Cur. In addition, we also found that the intracellular released of Cur subsequently upregulated the expression of DR4 and DR5 with increasing the apoptosis triggered by TRAIL.

## 2. Materials and methods

### 2.1. Materials, cell lines and animals

PEG (*M*_n_ = 4000, Sigma Aldrich, St. Louis, MO, USA), ε-caprolactone (ε-CL, Alfa Aesar, Ward Hill, MA, USA), stannous octoate (Sn(Oct)_2_, Aldrich), Cur (Sigma Aldrich, St. Louis, MO, USA, the purity more than 98%) and methyl thiazolyl tetrazolium (MTT, Sigma Aldrich, St. Louis, MO, USA) were performed without further purification. The extracellular domain of TRAIL (amino acids 114–281) was provided from Chengdu Huachuang Biotechnology Co. Ltd. HOOK^TM^-Dye Labeling Kit (G-Biosciences, St Louis, MO, USA) was used to label TRAIL with fluorescent dyes. Anti-Ki67 antibody was purchased by Thermo Scientific, anti-DR4 and anti-DR5 antibodies were provided from Abcam, and caspase-3 and PARP antibody were obtained from Cell Signaling. Cell apoptosis was detected using TdT-mediated Dutp nick-end labeling (TUNEL) staining (in situ cell death detection kit, Madison, WI).

HCT116 colon cancer cells were incubated in 5% CO_2_ atmosphere with McCoy 5 A (Gibco, Invitrogen, Grand Island, NY, USA) media supplemented with 10% fetal bovine serum (FBS, Gibco) at 37 °C. Trypsin-EDTA (0.25%) was applied for cell passaging.

Male nude mice were housed in groups of five, kept under pathogen-free conditions, fed with food and water ad libitum and acclimatized for a week. This study was granted by the Ethics Committee on Animal Experimentation of Sichuan University (Chengdu, China).

### 2.2. Synthesis of copolymer

We synthesized PCEC copolymer with molecular weight of 12,000 by ring-opening polymerization of ε-CL and PEG (*M*_w_ = 4000) using Sn(Oct)_2_ as catalyst (Gou et al., 2009b). The gained PCEC copolymer was purified and stored in desiccators for further application.

### 2.3. Preparation and characterization of drug-loaded nanoparticles

We used modified emulsion solvent evaporation method to form codelivery nanoparticles. In brief, 2 mL of Cur solution (dissolved in acetone, 3 mg/mL) and 2 mL of PCEC solution (dissolved in ethyl acetate, 20 mg/mL) were dropped into 8 mL of aqueous solution containing 0.5 mg of SDS under stirring at speed of about 12,000 rpm using a rotor-stator device (T10, IKA, German). After 8 min, the O/W emulsion was produced and then put in rotor evaporator flask. Cur-loaded nanoparticles (Cur-NPs) were formed using the convenient and efficient rotor evaporation (BUCHI, Switzerland). The Cur-NPs were dialyzed in PBS for 3 h to remove free SDS. The excess drugs were filtrated via a 0.22-μm syringe filter (Millex-LG, Millipore Co., Billerica, MA, USA). Subsequently, 0.2 mL of TRAIL solution (5 mg/mL) was added into 1 mL of Cur-NPs under magnetic stirring to form TRAIL-Cur-NPs.

Malvern Nano-ZS 90 laser particle size analyzer was used to detect the size distribution and zeta potential of drug-loaded nanoparticles. A transmission electron microscopy (TEM, H-6009IV, Hitachi, Japan) was applied to investigate the morphology of the TRAIL-Cur-NPs.

Cur-loading efficiencies in Cur-NPs and TRAIL-Cur-NPs were detected by high-performance liquid chromatography (HPLC, Waters Alliance 2695) (Yang et al., 2015). The amount of TRAIL loaded in nanoparticles was investigated using BCA protein kit as previously described (Kim et al., 2013). Drug loading (DL) and encapsulation efficiency (EE) were analyzed using the following Equations (1,2).

(1) DL (%)=(mass of drug in the sample/mass of sample)×100%

(2) EE (%)=(experimental drug loading/theoretical drug loading)×100%

### 2.4. *In vitro* drug release behavior

A modified dialysis method was performed to evaluate the *in vitro* release behavior of Cur-loaded nanoparticles according to our previous reports (Yang et al., 2015). Cur and TRAIL concentration was detected though the methods mentioned above. All samples were formulated and investigated in triplicate.

### 2.5. *In vitro* cellular uptake

Rhodamine-mark TRAIL (Rho-TRAIL) was used in the cellular uptake assay. To obtain Rho-TRAIL, dye (5/6)-TAMRA-labeling reagent dissolved in DMSO were slowly dropped into TRAIL solution (molar ratio = 6:1, V/V = 1:100), and then, the reaction mixture was kept at room temperature for 60 min. Subsequently, we used the SpinOUT^TM^ GT-600 column to remove the unconjugated dye and purify labeled proteins following the instructions. Lastly, the purified Rho-TRAIL was bound on the anionic Cur-NPs under magnetic stirring to form Rho-TRAIL-Cur-NPs by electrostatic interactions.

Cellular uptake of TRAIL-Cur-NPs was assessed by fluorescence microscopy and flow cytometric (FCM) analysis. HCT116 cells at a density of 2 × 10^5^ cells/mL were seeded into six-well plates with glass coverslips and incubated in 2 mL of medium for 24 h. The attached cells were exposed to Rho-TRAIL-Cur-loaded nanoparticles (TRAIL-Cur-NPs) in serum-free medium. Rho-TRAIL-loaded nanoparticles (TRAIL-NPs), Cur-loaded nanoparticles (Cur-NPs), free Rho-TRAIL (TRAIL) and free Cur (Cur) with an equivalent dose were used as control. After cultivation for 2 h at 37 °C, the cells were rinsed twice with PBS, fixed with ice-cold acetone for 30 min and stained with DAPI. All the images gathered by fluorescence microscopy. Moreover, the attached cells were treated as present above and quantitatively evaluated by FCM.

### 2.6. *In vitro* cytotoxicity assay

HCT116 cells at a density of 2 × 10^3^ cells/well were cultured in 96-well plates. After incubated for 24 h, the cells were treated with TRAIL-Cur-NPs, TRAIL-loaded nanoparticles (TRAIL-NPs), Cur-NPs, free TRAIL plus Cur (TRAIL + Cur), free nanoparticles (NPs), free TRAIL (TRAIL) and free Cur (Cur) at different concentrations for another 24 h. Subsequently, 20 μL of 3-(4, 5-dimethylthiazol-2-yl)-2, 5-diphenyltetrazolium bromide (MTT) solution was dropped to each well. After 4 h incubated, crystals were melted by dimethyl sulfoxide (DMSO), and then, the absorbance was measured at 570 nm. The data were determined in six measurements as mean value ± SD.

### 2.7. Apoptosis evaluation

Apoptosis was evaluated using a sub-G1 fraction. Approximately 1 × 10^6^ HCT116 cells were cultured in six-well plates overnight. After treatment with various treatments for 24 h, cells were harvested, washed twice with cold PBS and fixed with 70% ethanol for 30 min. Then, samples were exposed to RNAse A for 40 min, stained with propidium iodide (PI, 30 mg/mL) at 4 °C for 30 min and measured with FCM (FACS Calibur, BD). The fluorescence excitation was supplied by a 488 nm argon laser beam, and emission fluorescence was evaluated larger than 630 nm. Sub-G1 (apoptotic) cells/total number of cells were calculated by the software of NovoExpress. After treatment with different groups as described above, the expression of caspase 3, cleaved caspase 3, PARP and cleaved PARP at protein levels was investigated using Western blot.

### 2.8. Biodistribution of TRAIL-cur-NPs

The HCT116 colon cancer xenograft model was established by implanting 1 × 10^7^ tumor cells into the subcutaneous tissue of the right flank. To evaluate the real-time distribution and tumor accumulation efficiency, we used Coumarin-6 (Cou) to take the place of Cur following by incubation with Rho-TRAIL. Then, the fluorescence-labeled nanoparticles system was formed (Rho-TRAIL-Cou-NPs). When tumors reached 500 mm^3^ in size, the tumor-bearing mice were injected intravenously with free Rho-TRAIL, Rho-TRAIL-NPs, Cou-NPs and Rho-TRAIL-Cou-NPs at the same dosage (5 mg/kg Rho-TRAIL and/or 5 mg/kg Cou dosage). The fluorescent scans were performed at different time points (2, 6, 12 and 24 h), and subsequently, the mice were sacrificed. The main organs (lung, heart, liver, kidney, spleen and tumor) were dissected, and the fluorescent images were investigated by fluorescence image system (Quick view 3000, Bio-Real, Austria). The fluorescence signals of Cou (*E*_x_ = 474 nm, *E*_m_ = 520 nm) and Rhodamin (*E*_x_ = 535 nm, *E*_m_ = 670 nm) were recorded, respectively.

### 2.9. *In vivo* tumor inhibition assay

Five to six weeks male BALB/c nude mice (17 ± 2 g) were used to establish HCT116 colon cancer xenograft models by implanting HCT116 cells (1 × 10^7^) into the right flank. When the size of tumor reached 200 mm^3^ (day 0), mice were randomly divided into eight groups (*n* = 6) and intravenously injected with control (5% glucose solution), NPs, free TRAIL, TRAIL-NPs, free Cur, Cur-NPs, TRAIL + Cur and TRAIL-Cur-NPs at an equivalent dose (30 mg/Kg TRAIL and/or 30 mg/kg Cur) on selected days (0, 2, 4, 6, 8 and 10 day). Body weight and tumor volume were observed every three days. Tumor volume was calculated by *ab*^2^/2, in which *a* and *b* mean the longest and shortest diameters of the tumor.

### 2.10. Immunohistochemically analysis of tumors

The excised tumors were fixed in formalin, embedded in paraffin and sectioned into slices at a thickness of 5 μm for further immunohistochemically staining. TUNEL and Ki-67 staining were performed to assess the tumor cell apoptosis and proliferation, respectively. To study the mechanism of antitumor activity in mice, the expression of DR4 and DR5 were determined by immunohistochemistry. Moreover, five images of each sample group were collected to quantitatively measure by Image-Pro Plus (Media Cybernetics Inc., Rockville, MD, USA).

### 2.11. Gene and protein expression of death reporters in tumors

Total RNA was isolated from frozen xenograft tumor specimens in all treated groups using the Trizol Reagent (Invitrogen, Grand Island, NY, USA). The cDNA was produced by Super RT cDNA kit (CWBio, Beijing, China) following the instructions. RT-PCR was performed using RealMasterMix (SYBR Green, TIANGEN, Beijing, China). Relative gene expression was established according to the 2^(−ΔΔCT)^ method.

The tumor tissues of all groups were dislocated by sonication and extracted at 4 °C for 30 min. Whole-cell protein lysates were separated by electrophoresis through a 12% SDS-PAGE gels, and then, proteins were transported onto a polyvinylidene fluoride membrane (PVDF). The membrane was incubated with primary antibodies including DR5 (1:50,000, Abcam, Cambridge, MA, USA), DR4 (1:1000, Abcam) and GAPDH (1:2000, ZSGB-BIO, Beijing, China). Primary antibodies were exposed using goat anti-rabbit horseradish peroxidase-link secondary antibodies (1:5000, SANTA CRUZ BIOTECHNOLOGY, Dallas, TX, USA). The detection of specific proteins was exposed to Kodak-X-Omat films carried out using ECL Western Blotting Substrate (Thermo Fisher Scientific, Waltham, MA, USA). 

### 2.12. Toxicity assessment

To estimate the side effects in each group, tissue sample and blood sample were collected and used for hematology test and serum chemistry profile test. Tissue histopathology of major organs were obtained, fixed in 4% paraformaldehyde in PBS for 48 h, and stained with H&E.

### 2.13. Statistical analysis

Data were given as mean ± SD of independent experiments. The significance among the samples was evaluated using one-way analysis of variance (ANOVA). Values were considered statistically significant starting from *p* < .05. Statistics were calculated by SPSS (version 19, SPSS Inc., Chicago, IL, USA).

## 3. Results

### 3.1. Preparation and characterization of TRAIL-cur-NPs

In this work, the hydrophobic drug Cur was encapsulated into the inner core of PCEC nanoparticles to form Cur-NPs by modified emulsion solvent evaporation method. Subsequently, the positively charged TRAIL with a molecular weight of about 19 kDa was attached on the surface of the negatively charged nanoparticles via charge interaction (Figure S1 (A)). TRAIL-NPs and Cur-NPs were prepared in similar methods as described above. In Figure S1 (B), a spheroid structure of TRAIL-Cur-NPs was observed by the transmission electron microscope (TEM). The TRAIL-Cur-NPs have a narrow particle size distribution with an average diameter of 143 ± 5 nm and a polydispersity index (PDI) of 0.16 detected by DLS (Figure S1 (C)). After TRAIL attached to the surface of Cur-NPs forming TRAIL-Cur-NPs, a slight increase in the zeta potential from −13.6 ± 0.8 mV to −10.7 ± 0.6 mV (Figure S1 (D)) was observed. DL of TRAIL and Cur in TRAIL-Cur-NPs were 9.81 ± 0.11% (with EE of 99.26 ± 1.22%) and 9.75 ± 0.06% (with EE of 96.36 ± 2.21%), respectively (Table S1). As shown in Figure S2 (A), the appearance of TRAIL-Cur-NPs implied that the nanoparticles could well dispersed in aqueous solution. Consequently, loading TRAIL and Cur into TRAIL-Cur-NPs could lead to a homogenous and stable dosage form in aqueous media codelivering these two drugs *in vivo* by intravenous injection.

In the *in vitro* drug release experiments, the release behaviors of TRAIL and Cur from nanoparticles were continuously monitored in 14 days. Both free TRAIL and free Cur displayed rapidly release behavior compared to slow cumulative release rate of TRAIL-Cur-NPs. As shown in Figure S2 (B), 48.22% of TRAIL was released from TRAIL-Cur-NPs in the first 48 h, and approximately, 85.25% of TRAIL was released within 336 h. About 23.13% and 44.29% of Cur were released from TRAIL-Cur-NPs within 48 h and 336 h, respectively. However, approximately, 86.65% and 100% of Cur were quickly released in free Cur group within 48 h and 336 h, respectively.

### 3.2. TRAIL improved cellular uptake of nanoparticles *in vitro*

**Figure 1.**
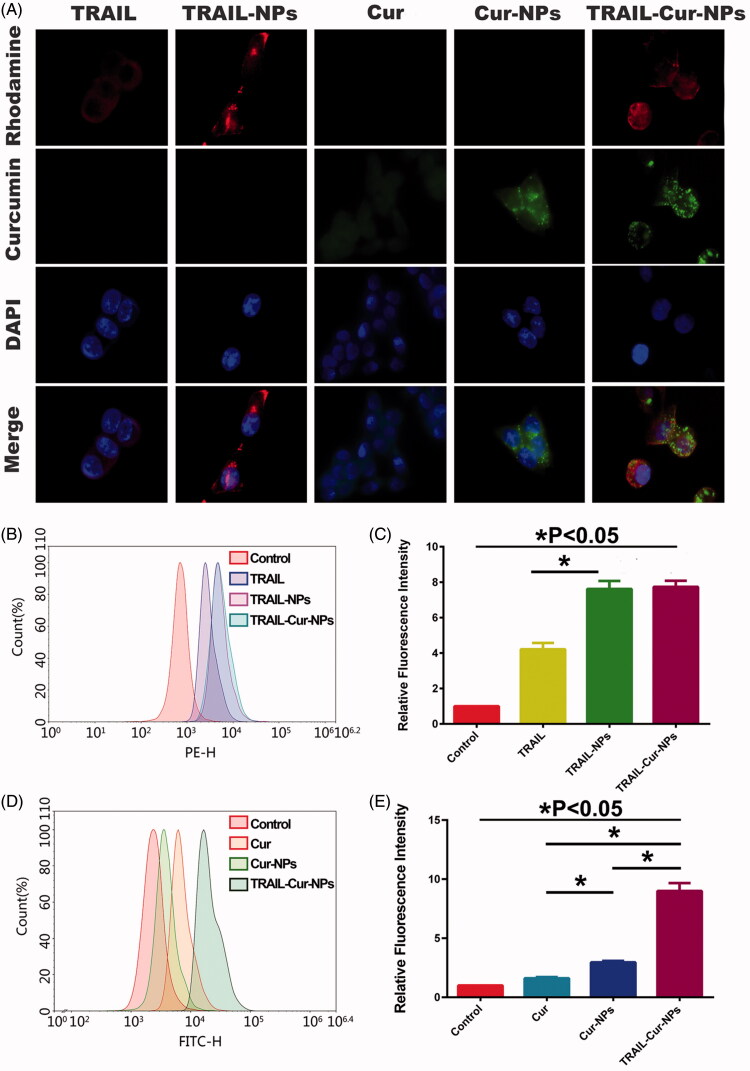
Cellular uptake study of TRAIL-Cur-NPs in HCT116 cells. (A) Fluorescent images of cells exposed to TRAIL, TRAIL-NPs, Cur, Cur-NPs and TRAIL-Cur-NPs for 2 h. (B) FCM analysis of cellular uptake in control, TRAIL, TRAIL-NPs and TRAIL-Cur-NPs for 2 h, respectively. (C) Mean fluorescence intensity in control, TRAIL, TRAIL-NPs and TRAIL-Cur-NPs, respectively. (D) FCM analysis of cellular uptake in control, Cur, Cur-NPs and TRAIL-Cur-NPs for 2 h, respectively. (E) Mean fluorescence intensity in control, Cur, Cur-NPs and TRAIL-Cur-NPs, respectively.

As illustrated in Figure 1(A), the fluorescence intensity of HCT116 in nanoparticles groups (TRAIL-NPs, Cur-NPs and TRAIL-Cur-NPs) were higher than that in free drug groups. Furthermore, cells treated with TRAIL-Cur-NPs showed higher intracellular accumulation of Cur than free Cur and Cur-NPs suggesting the enhanced cellular uptake via TRAIL-mediated endocytosis. In addition, the intracellular delivery of TRAIL-Cur-NPs in HCT116 cells was investigated by FCM. The quantitative analysis of cellular uptake was in agreement with the observation from fluorescence microscopy. In Figure 1(B,C), the relative fluorescence intensity of TRAIL-NPs and TRAIL-Cur-NPs was higher than that in the free TRAIL, and much stronger green fluorescence was detected in TRAIL-Cur-NPs than that in Cur-NPs or free Cur (Figure 1(D,E)). Therefore, the results demonstrated that TRAIL could enhance the cellular uptake of TRAIL-Cur-NPs.

### 3.3. *In vitro* cytotoxicity and apoptosis

**Figure 2.**
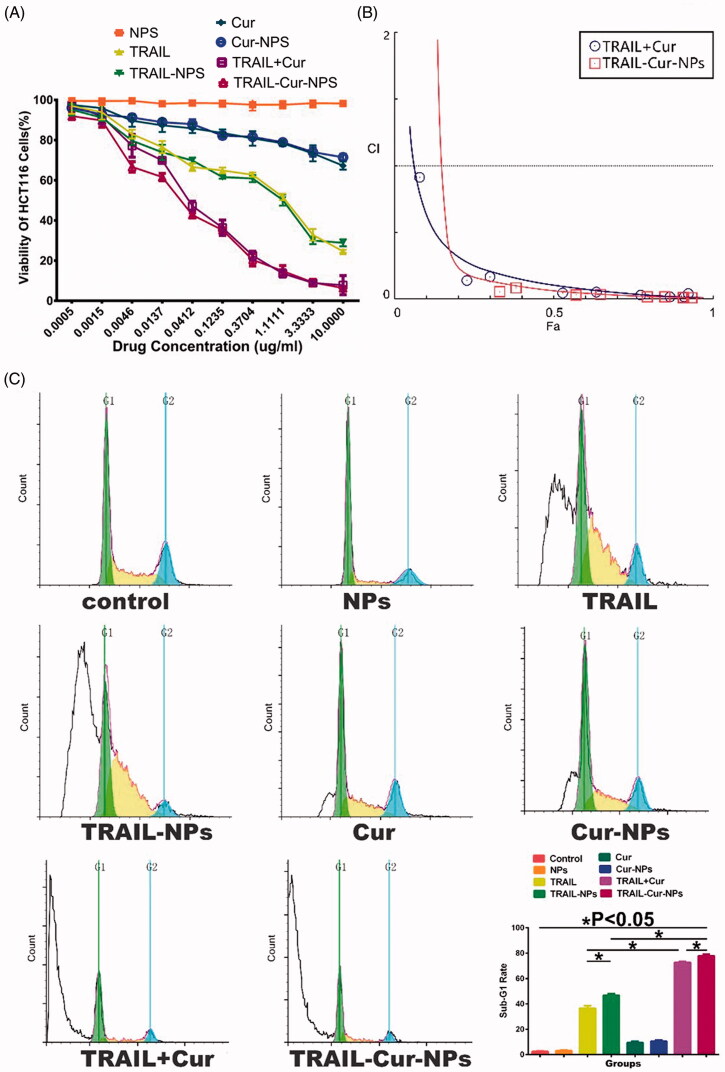
*In vitro* cytotoxicity and apoptosis. (A) *In vitro* cytotoxicity evaluation of different groups toward HCT 116 cells for 24 h. (B) Combination index curves of TRAIL-Cur-NPs and free TRAIL + Cur. (C) Flow cytometric analysis of apoptosis on HCT 116 cells using PI staining.

As illustrated in Figure 2(A), *in vitro* cytotoxicity of TRAIL-Cur-NPs against HCT116 cells was evaluated by MTT assay at 24 h. Blank nanoparticles showed negligible toxicity in all the tested concentrations. Free Cur and Cur-NPs only showed a slight inhibition effect on the cells, and both TRAIL and TRAIL-NPs exhibited moderate cytotoxicity. Moreover, both TRAIL-Cur-NPs and TRAIL + Cur showed much stronger antitumor effect than those monotherapies implying a synergetic effect of TRAIL and Cur. Although TRAIL-Cur-NPs group exhibited almost the same cytotoxicity with TRAIL + Cur, the half-maximal inhibitory concentration (IC_50_) of TRAIL-Cur-NPs was 0.28 μg/mL, which lower than that of TRAIL + Cur (0.66 μg/mL). These results suggested that compare with free drugs, codelivery TRAIL and Cur by TRAIL-Cur-NPs improved the cytotoxicity on tumor cells. To further study, the synergetic effect of TRAIL and Cur, combination index (CI) determination was carried out. The CI values lower than 1 indicated synergism, higher than 1 demonstrated antagonism, and equal to 1 denoted additivity (Zhao et al., 2004). Both TRAIL + Cur and TRAIL-Cur-NPs exhibited strong synergism with CI values less than 0.3 (Figure 2(B)).

We used FCM assay to measure the apoptosis-inducing activity of TRAIL-Cur-NPs by observing sub-G1 (apoptotic) cells. As indicated in Figure 2(C), free Cur and Cur-NPs (Cur concertation 300 ng/mL) showed similarly low apoptosis ratio about 10%. The apoptotic ratio of free TRAIL and TRAIL-NPs was 36.52 ± 2.10% and 44.66 ± 1.21% (*p* < .05), respectively. Moreover, both TRAIL-Cur-NPs and free TRAIL + Cur showed significant increase in apoptosis, suggesting the synergistic apoptosis-inducing effect of TRAIL and Cur. TRAIL-Cur-NPs induced more apoptosis than free TRAIL + Cur (77.82 ± 1.22% vs. 72.60 ± 0.82%, *p* < .05). Additionally, as shown in Figure S3, the protein expression of cleaved caspase-3 and cleaved PARP in TRAIL-Cur-NPs group were slightly stronger than those in free TRAIL + Cur group in HCT116 cells. In summary, TRAIL-Cur-NPs could enhance much more apoptosis of HCT116 cells than that of the free TRAIL + Cur.

### 3.4. *In vivo* tumor targeting

**Figure 3.**
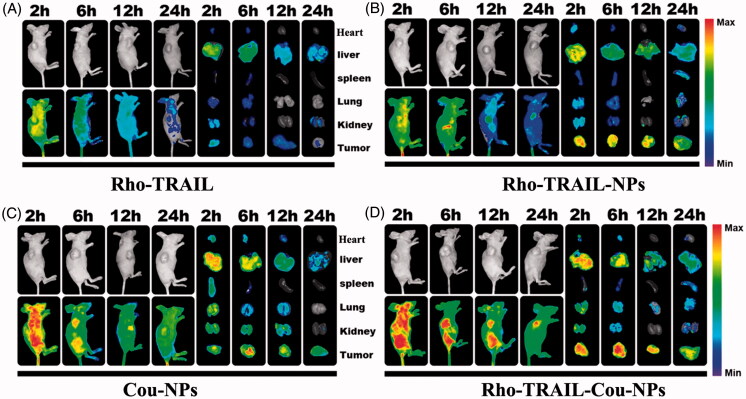
*In vivo* fluorescence imaging of the HCT 116 tumor-bearing nude mice at 2, 6, 12 and 24 h after intravenous injection. (A) Rho-TRAIL, (B) Rho-TRAIL-NPs, (C) Cou-NPs, (D) Rho-TRAIL-Cou-NPs.

To detect the tumor targeting of the codelivery nanoparticles *in vivo*, Rho-TRAIL and Cou were coloaded in the nanoparticles (Rho-TRAIL-Cou-NPs). Free Rho-TRAIL, Rho-TRAIL-NPs and Cou-NPs were used as control. As indicated in Figure 3(A,B), Rho-TRAIL-NPs displayed a stronger fluorescence signal at the tumor sites after 2 h than that in free Rho-TRAIL group. As time extended, a much slower fluorescence signal attenuation in the tumor regions was observed in Rho-TRAIL-NPs group compared to free Rho-TRAIL. To further prove the role of Rho-TRAIL in the active targeting of Rho-TRAIL-Cou-NPs, *in vivo* biodistribution of Cou-NPs and Rho-TRAIL-Cou-NPs were evaluated. As illustrated in Figure 3(C,D), Rho-TRAIL-Cou-NPs exhibited more drug accumulation at the tumor sites compared to Cou-NPs, suggesting that TRAIL endowed the nanoparticles with active tumor-targeting ability. Furthermore, little fluorescence signal aggregated in normal tissues, which indicated the efficient tumor targeting effects and fewer side effects of the codelivery nanoparticles.

### 3.5. *In vivo* antitumor efficacy

**Figure 4.**
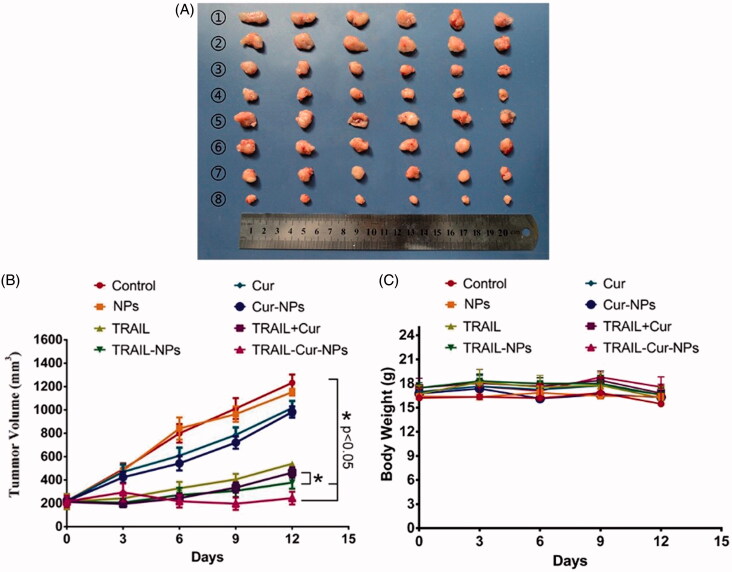
Antitumor efficacy in subcutaneous HCT116 model. (A) Tumors from different treatment groups: ① Control, ② NPs, ③ free TRAIL, ④ TRAIL-NPs, ⑤ free Cur, ⑥ Cur-NPs, ⑦ TRAIL + Cur, ⑧ TRAIL-Cur-NPs. (B) The tumor growth curves of different groups in tumor-bearing mice. (C) Changes of mouse body weight in different groups.

*In vivo* antitumor efficacy of TRAIL-Cur-NPs was explored on human colon carcinoma HCT116 xenograft model. All the treatment groups (control, blank NPs, free TRAIL, TRAIL-NPs, free Cur, Cur-NPs and TRAIL + Cur and TRAIL-Cur-NPs) were administrated at the same dose of drugs (30 mg/kg equal of TRAIL and/or 30 mg/kg Cur). As illustrated in Figure 4(A,B), the tumor volume of various TRAIL-treated groups was significant lower than control, blank NPs, free Cur and Cur-NPs group (1232.59 ± 70.57 mm^3^, 1152.13 ± 28.95 mm^3^, 1016.51 ± 59.91 mm^3^ and 982.15 ± 48.64 mm^3^, respectively). Moreover, TRAIL-Cur-NPs induced a noticeably higher inhibiting effect on tumor growth (151.10 ± 46.12 mm^3^) than free TRAIL + Cur group (463.69 ± 44.68 mm^3^, *p* < .05) due to the higher drug accumulation in the tumor regions via the passive targeting of nanoparticles and active targeting capability provided by TRAIL. Furthermore, the body weight of mice remained stable during experimental period indicating no significant side effects in all groups (Figure 4(C)).

We evaluated the proliferation activity and apoptosis level in the tumors from different groups by immunohistochemically staining of Ki-67 and TUNEL. Compare with the other treatment groups, the results of Ki-67 staining treated with TRAIL-Cur-NPs showed remarkably lower tumor cell proliferation in subcutaneous HCT116 model (Figure S4). As shown in Figure S5, TUNEL staining assays revealed that TRAIL-Cur-NPs induced more apoptosis than other groups. Therefore, these results suggested that TRAIL-Cur-NPs had a superior antitumor activity compared to other groups owing to inhibition of tumor proliferation and induction of tumor apoptosis.

### 3.6. Death receptors expression

**Figure 5.**
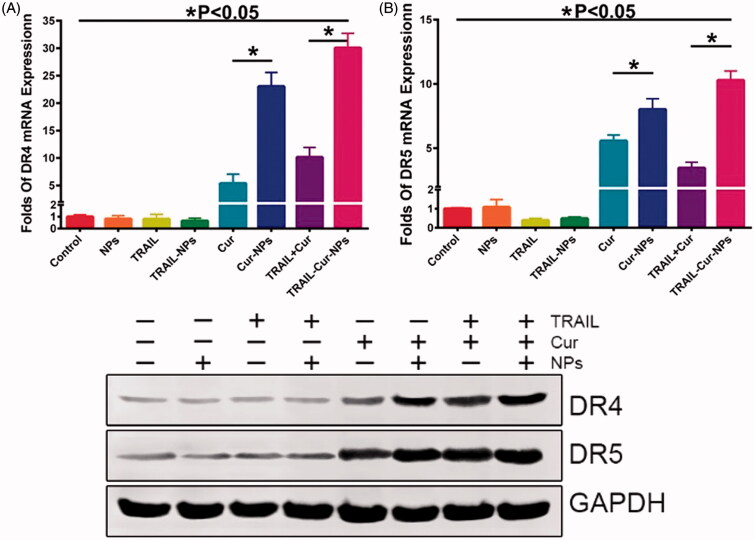
Expression of death receptors in tumor tissue. (A) The mRNA expression of DR4. (B) The mRNA expression of DR5. (C) Western blot analysis of DR4 and DR5 protein levels.

Previous studies submitted that the synergistic effect of TRAIL and Cur may be responsible for the induction of DR5 expression (Jung et al., 2005, 2006). To investigate the synergistic effect, RT-PCR, western blot and immunohistochemically staining for DR4 and DR5 were used to assess the gene and protein expression of DR4 and DR5 in tumors. As indicated in Figure 5(A), DR4 gene expression was suppressed in TRAIL-treated groups while increased in Cur-treated groups (*p* < .05). Similarly, DR5 gene expression of free TRAIL and TRAIL-NPs in Figure 5(B) were much lower than those in Cur, Cur-NPs, TRAIL + Cur and TRAIL-Cur-NPs (*p* < .05). Moreover, the protein expression of DR4 and DR5 were weaker in TRAIL-treated groups but stronger in Cur-treated samples in Figure 5(C).

Immunohistochemically analysis was used to further evaluate death receptors at tumor sites. As shown in Figure S6 and S7, significantly high protein expression of DR4 and DR5 were examined in tumor tissues with Cur-treated groups compared with TRAIL-treated groups. Moreover, the DR4 and DR5 expression rate in TRAIL-Cur-NPs were significantly higher than other treatment groups (*p* < .05). Hence, these results further confirmed that the synergistic therapeutic efficacy of TRAIL and Cur in TRAIL-Cur-NPs group resulting from the upregulation of DR4 and DR5 induced by Cur.

### 3.7. *In vivo* toxicity evaluation

During the experiment period, no directly death and obvious side effects were observed. No significantly weight lost were detected among all groups (Figure 4(C)). To evaluate possible side effect in different groups, we performed serum chemistry profile test, complete blood count and histological evaluation of major organs. As illustrated in Figures S8 and S9, the outcomes of serum chemistry profile test and complete blood count showed no significantly difference between control and treatment groups (*p* > .05). In addition, the results of H and E staining of heart, spleen, lung, liver and kidney confirmed that there was no obvious toxicity in all the test groups (Figure S10).

## 4. Discussion

Since identified in 1995 by Wiley et al., TRAIL has been greatly impressed by scientists for its unique antitumor properties (Wiley et al., 1995; Ashkenazi, 2002). Although TRAIL-induced apoptosis of different kinds of human tumor cells without affecting normal cells, the short half-life, low *in vivo* therapeutic efficacy and drug resistance compromised its further applications. Now, therapeutic strategies of TRAIL-loaded nanocarriers mainly focus on improving the half-life and antitumor efficiency of TRAIL. For example, Lim et al. confirmed that TRAIL-loaded nanoparticles (NPs) made by the ionic interaction between polymer and TRAIL to improve half-life and enhance tumor cell apoptosis (Rivoltini et al., 2016). Kim et al. reported that TRAIL could be attached to the surface of PLGA microparticles by double emulsification method for lung cancer therapy (Kim et al., 2013). Jiang group found that PLGA microspheres loaded with PEGylated TRAIL and doxorubicin (DOX) by double-emulsion solvent extraction method showed an encouraging anticancer efficacy against human colon cancer HCT116 *in vitro* and *in vivo* (Jiang et al., 2011). Moreover, the cationic micellar nanoparticles self-assembled from P(MDS-*co*-CES) were used to codeliver TRAIL and DOX for inhibiting the growth of TRAIL-resistant SW480 colorectal carcinoma (Lee et al., 2011). To the best of our knowledge, the prepared TRAIL-Cur-NPs in our manuscript is the first reported TRAIL and Cur codelivery nanoparticles. Compared to these existing TRAIL-coated nanocarriers, TRAIL-Cur-NPs based on biodegradable PCEC triblock copolymer may be advantageous due to following reasons (Guo et al., 2011, 2012; Lee et al., 2011; De Miguel et al., 2016a; Rivoltini et al., 2016; Pan et al., 2013). The ‘two-in-one’ strategy used to load Cur and TRAIL in TRAIL-Cur-NPs could efficiently improve the therapeutic effect of the drugs by prolonging their circulation time, enhancing the water dispersion of Cur and improving the active and passive tumor targeting. In TRAIL-Cur-NPs, TRAIL acted as both active targeting ligand and therapeutic agent, while Cur could increase death receptors expression to enhance the antitumor effect. In addition, both PEG and PCL, biocompatible and biodegradable polymer, have been used to several US FDA-approved products (Gou et al., 2009a, 2009b; Zhang et al., 2012). The results of safety evaluation indicated that the PCEC nanoparticles could be a safe candidate as drug delivery system *in vitro* and *in vivo* (Wei et al., 2009). Taken together, TRAIL-Cur-NPs have several important advantages to improve efficacy without toxicity. However, there are still many challenges associated with controllable, reproducible and scalable TRAIL-Cur-NPs preparation, as well as long-term preservation of TRAIL-Cur-NPs, which need to be overcome to achieve the success in clinical application.

**Figure 6.**
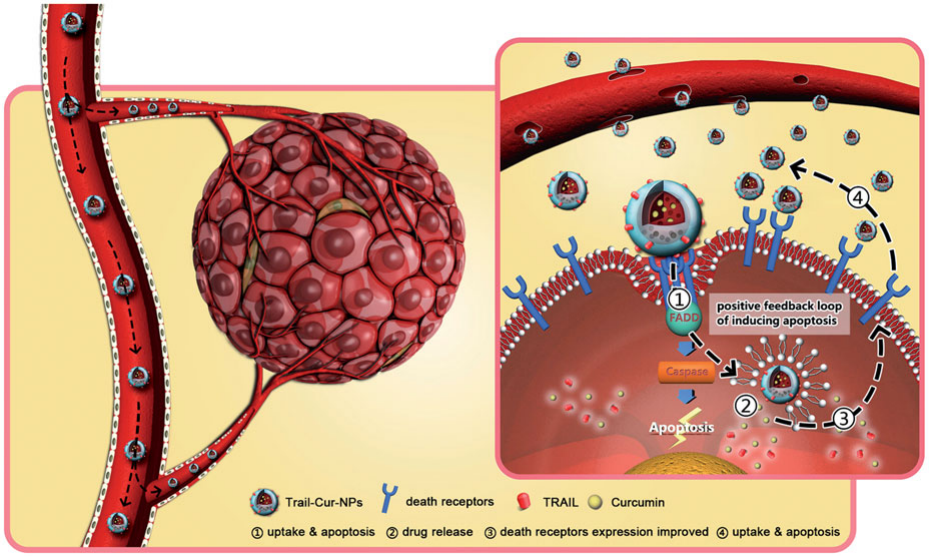
Schematic illustration of *in vivo* antitumor mechanism of TRAIL-Cur-NPs. The prepared TRAIL-Cur-NPs were accumulated in extracellular tumor tissue though the enhanced permeability and retention (EPR) effect. TRAIL on the surface of TRAIL-Cur-NPs triggered on the death receptors (DR4 and DR5) and recruited the adaptor molecule Fas-associated death domain-containing protein (FADD). Then, the death domain of FADD bond to caspase-8 for the formation of a death-inducing signaling complex (DISC), which activated apoptotic pathways. More importantly, TRAIL also enhanced cellular uptake of nanoparticles via interaction between death receptors and TRAIL. Subsequently, the intracellular accumulation of Cur released by TRAIL-Cur-NPs could enhance the anti-tumor efficacy and the expression of death receptors, which improved the sensitivity of HCT116 to TRAIL. TRAIL-Cur-NPs established a positive feedback loop to induce apoptosis of cancer cells.

Cur has a broad range of pharmacological properties particularly anticancer activity. Some previous studies demonstrated synergistic effect of TRAIL and Cur in difference cancer cells (Jung et al., 2005; Deeb et al., 2007; Andrzejewski et al., 2008). DR5 was distributed on the cell surface and in the cytoplasm when it was induced by Cur (Yamanaka et al., 2000; Naka et al., 2002; James et al., 2015). Jung et al. suggested that the DR5 played a vital role in synergistic cytotoxicity associated with TRAIL and Cur (Jung et al., 2006). In addition, the augmentation of TRAIL-induced apoptosis had been partly the results of Cur-induced upregulation of death receptors (Evdokiou et al., 2002; Singh et al., 2003; Bouralexis et al., 2004). Our results suggested that TRAIL-Cur-NPs could significantly enhance the expression of DR4 and DR5 both in mRNA and in protein levels (Figure 5). As shown in the schematic illustration of Figure 6, the prepared TRAIL-Cur-NPs were accumulated in extracellular tumor tissue though EPR effect. TRAIL on the surface of TRAIL-Cur-NPs triggered on the death receptors (DR4 and DR5) and recruited the adaptor molecule Fas-associated death domain-containing protein (FADD) (Bellail et al., 2010; Xu et al., 2013). Then, the death domain of FADD bond to caspase-8 for the formation of a death-inducing signaling complex (DISC), which activated apoptotic pathways. TRAIL also acted as active targeting ligand to enhance cellular uptake of nanoparticles. Subsequently, the intracellular accumulation of Cur released by TRAIL-Cur-NPs could improve the expression of death receptors, which increased the sensitivity of HCT116 to TRAIL and then enhanced the apoptosis. Hence, the synthetic mechanism of TRAIL-Cur-NPs was proved to be a positive feedback loop to induce apoptosis of cancer cells.

In the *in vivo* experiments, TRAIL-Cur-NPs group showed significant higher anti-tumor efficiency than free TRAIL + Cur group in subcutaneous HCT116 tumor model (*p < *.05), but in the *in vitro* cytotoxicity evaluation, TRAIL-Cur-NPs group exhibited almost the same cytotoxicity with free TRAIL + Cur group. The explanation of inconsistent between *in vitro* and *in vivo* antitumor data of TRAIL-Cur and TRAIL-Cur-NPs groups may as follows. In the *in vivo* experiments, compared with free TRAIL + Cur group, TRAIL-Cur-NPs prolonged their circulation time of TRAIL and Cur in the blood system and improved accumulation of both drugs in the tumor sites by active and passive targeting effect. Then, in TRAIL-Cur-NPs, TRAIL act as both active targeting ligand and therapeutic agent to enhance the cellular uptake of NPs and trigger the apoptotic pathways, while Cur could enhance the expression of death receptors, providing a positive feed-back loop to increase the antitumor effect. Therefore, in the *in vivo* experiments, TRAIL-Cur-NPs group showed significant higher antitumor efficiency than free TRAIL + Cur group in subcutaneous HCT116 tumor model. In the contrast, in the *in vitro* cytotoxicity experiment, both TRAIL-Cur-NPs and free TRAIL + Cur groups exerted their synergetic antitumor efficacy, because equal amount of TRAIL and Cur was added into the cells without the influence of different half-life in the blood circulation system and accumulation in the tumor sites. Thus, TRAIL-Cur-NPs group exhibited almost the same cytotoxicity with free TRAIL + Cur group in the *in vitro* experiment. Additionally, no significant changes were observed in body weight, serum markers and the H&E staining of major organs between TRAIL-Cur-NPs and control group. In summary, TRAIL-Cur-NPs could be a promising candidate for colon cancer therapy.

## 5. Conclusions

In this study, we developed active targeting nanoparticles to codeliver TRAIL and Cur for the treatment of HCT116 colon cancer. TRAIL-Cur-NPs showed excellent properties such as small particle size for intravenous application, sustained release behavior and increased cellular uptake. Moreover, TRAIL-Cur-NPs exhibited superior efficacy in inhibiting growth of HCT116 colon carcinoma *in vitro* and *in vivo* due to the tumor targeting and synergistic effect of TRAIL and Cur. We found that the synergistic mechanism of improving antitumor efficacy of TRAIL and Cur was the upregulation of DR4 and DR5 induced by Cur. Therefore, these results suggested that TRAIL-Cur-NPs might have promising applications in colorectal cancer therapy.

## Supplementary Material

IDRD_Gong_et_al_Supplemental_Content.doc
