# Supplementary material for: TRAIL and curcumin codelivery nanoparticles enhance TRAIL-induced apoptosis through upregulation of death receptors
Source: Drug Deliv. 2017 Oct 10;24(1):1526–36. doi: 10.1080/10717544.2017.1384863 (PMC8241104; doi:10.1080/10717544.2017.1384863)
Supplement: IDRD_Gong_et_al_Supplemental_Content.doc [file IDRD_A_1384863_SM2626.doc]

**Supporting Information**

**TRAIL and Curcumin Co-Delivery Nanoparticles Enhance TRAIL-induced Apoptosis through Upregulation of Death Receptors**

Xi Yang^1,+^, Zhaojun Li^1,2,+^, Qinjie Wu^1^, Shouchun Chen^3^, Cheng Yi^1^,* ,Changyang Gong^1^,*

^1^ Department of Medical Oncology, Cancer Center, State Key Laboratory of Biotherapy, West China Hospital, Sichuan University, China.

^2^ Department of Radiotherapy, Hainan General Hospital, Haikou, China.

^3^ Chengdu Huachuang Biotechnology Co. Ltd., Chengdu, China.

^+^ These authors contributed equally to this work.

Correspondence to: Changyang Gong (Telephone: +86-28-85164063; Fax: +86-28-85164060; Email: chygong14@163.com) or Cheng Yi (Telephone: +86-28-85164063; Fax: +86-28-85164060; Email: yicheng6834@163.com).

**Table S1.** Characterization of drug-loaded nanoparticles

| Groups | size (nm) | PDI | Zeta-potential (mV) | DL (%) | EE (%) |
| --- | --- | --- | --- | --- | --- |
| Blank-NPs | 147 ± 9 | 0.12 ± 0.03 | -14.4 ± 1.1 | - | - |
| TRAIL-NPs | 160 ± 4 | 0.18 ± 0.03 | -10.5 ± 0.8 | 9.83 ± 0.13 | 98.26 ± 0.80 |
| Cur-NPs | 168 ± 5 | 0.18 ± 0.02 | -13.6 ± 0.8 | 9.74 ± 0.19 | 96.53 ± 0.75 |
| TRAIL-Cur-NPs | 143 ± 5 | 0.16 ± 0.01 | -10.7 ± 0.6 | 9.81 ± 0.11 ^a^  9.75 ± 0.06 ^b^ | 99.26 ± 1.22 ^a^  96.36 ± 2.21 ^b^ |

PDI: polydispersity index; DL: drug loading; EE: encapsulation efficiency.

a：The DL and EE of TRAIL in nanoparticles.

b：The DL and EE of Cur in nanoparticles.

Values are reported as means ± SD (n=6).


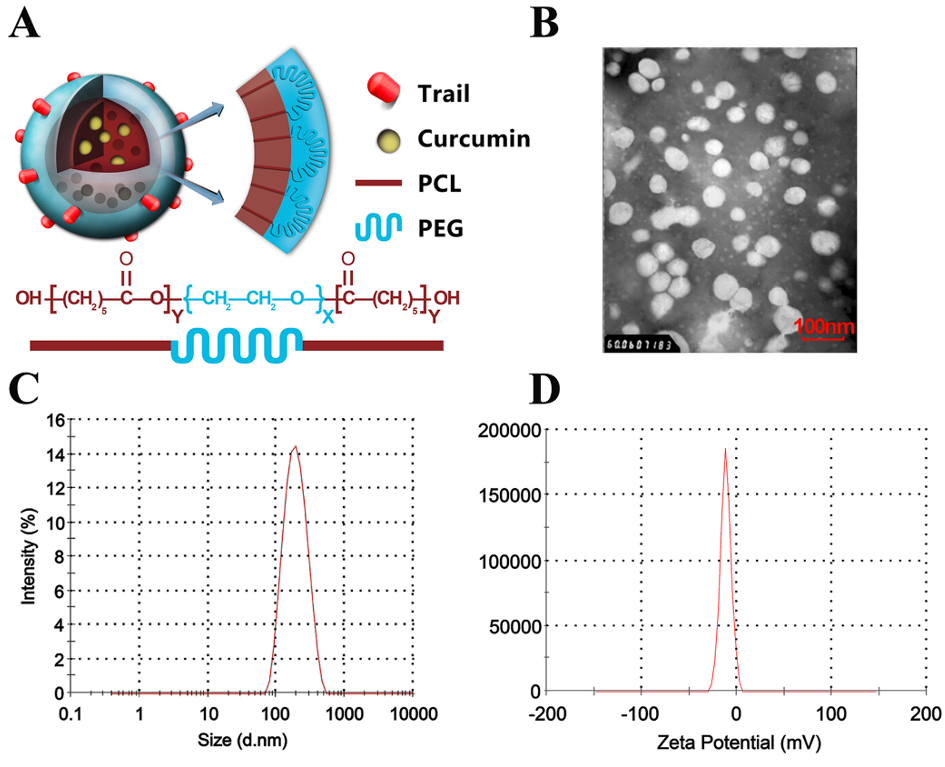


**Figure S1.** Preparation and characterization of TRAIL-Cur-NPs. (A) Schematic illustration of TRAIL-Cur-NPs. (B) TEM image of TRAIL-Cur-NPs. (C) & (D) Particle size and Zeta potential of TRAIL-Cur-NPs.


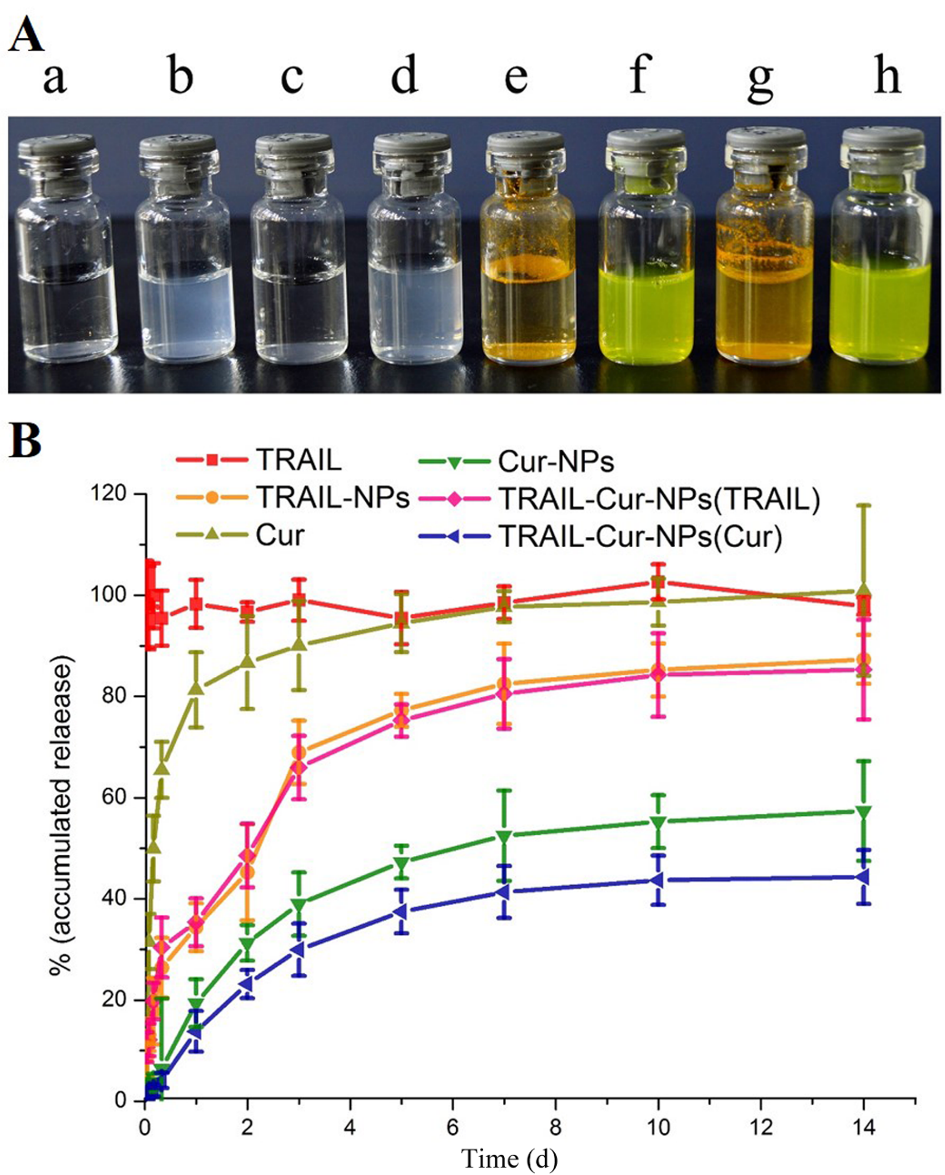


**Figure S2.** (A) Appearance of different groups: a) water, b) blank NPs, c) TRAIL in 5% glucose solution, d) TRAIL-NPs, e) free Cur in water, f) Cur-NPs, g) TRAIL + Cur in water, h) TRAIL-Cur-NPs. (B) The *in vitro* release profiles in each group (n = 4).


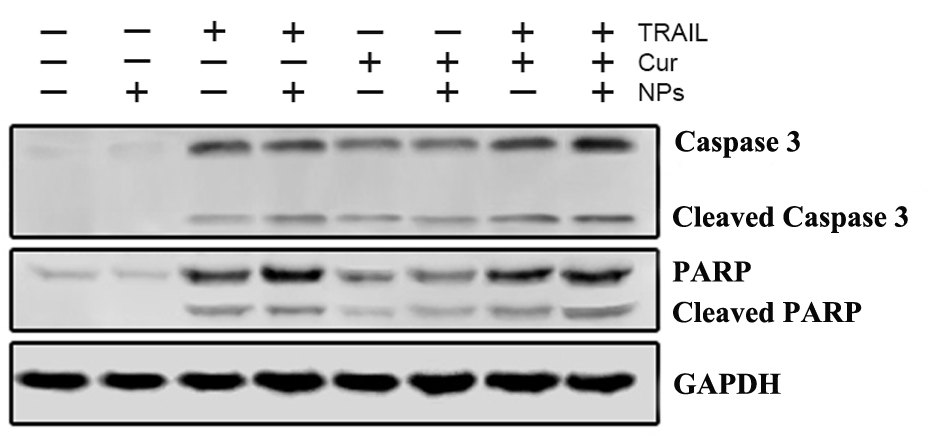


**Figure S3.** Western blot analysis of caspase 3, cleaved caspase 3, PARP, and cleaved PAPR protein levels in HCT116 cells.

**
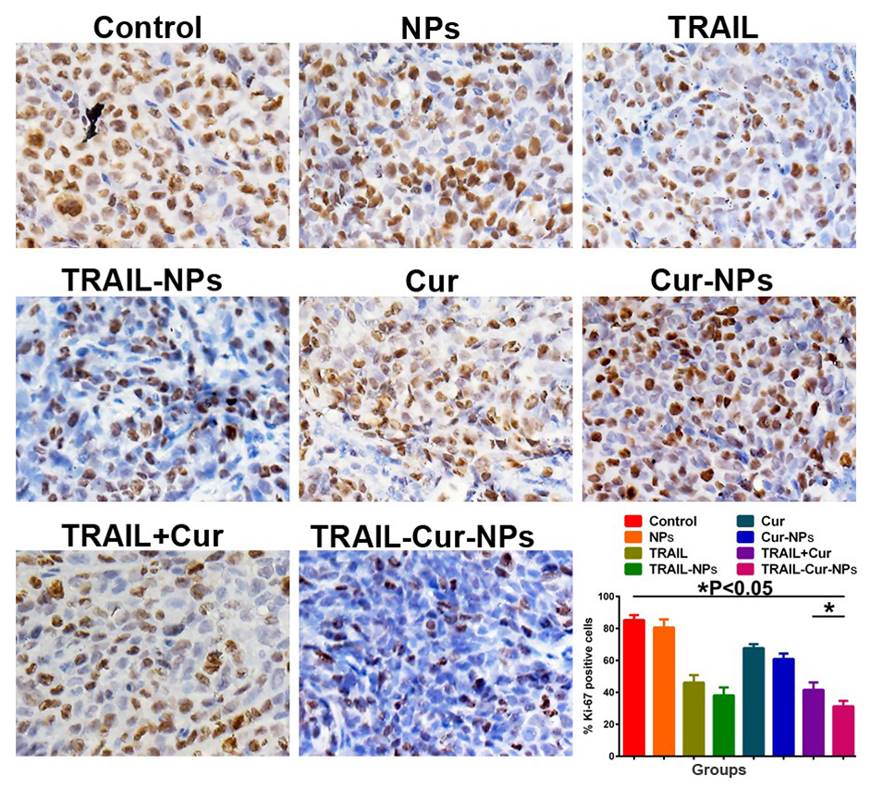
**

**Figure S4.** Ki-67 immunohistochemically staining of HCT116 tumors in different treatment groups.

**
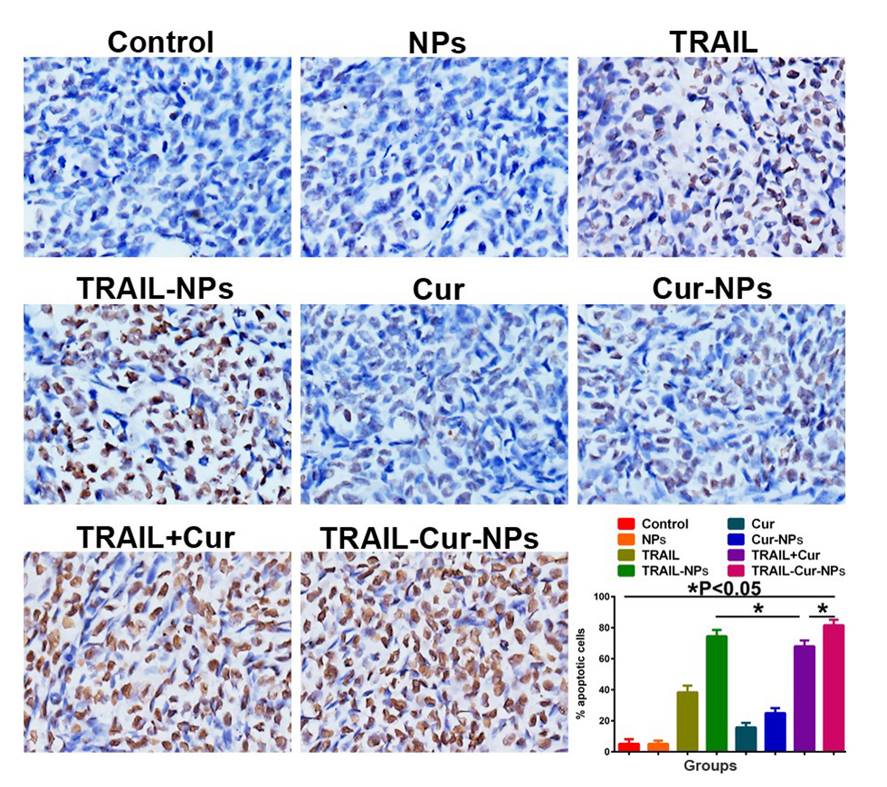
**

**Figure S5.** TUNEL analysis of HCT116 tumors in different treatment groups.

**
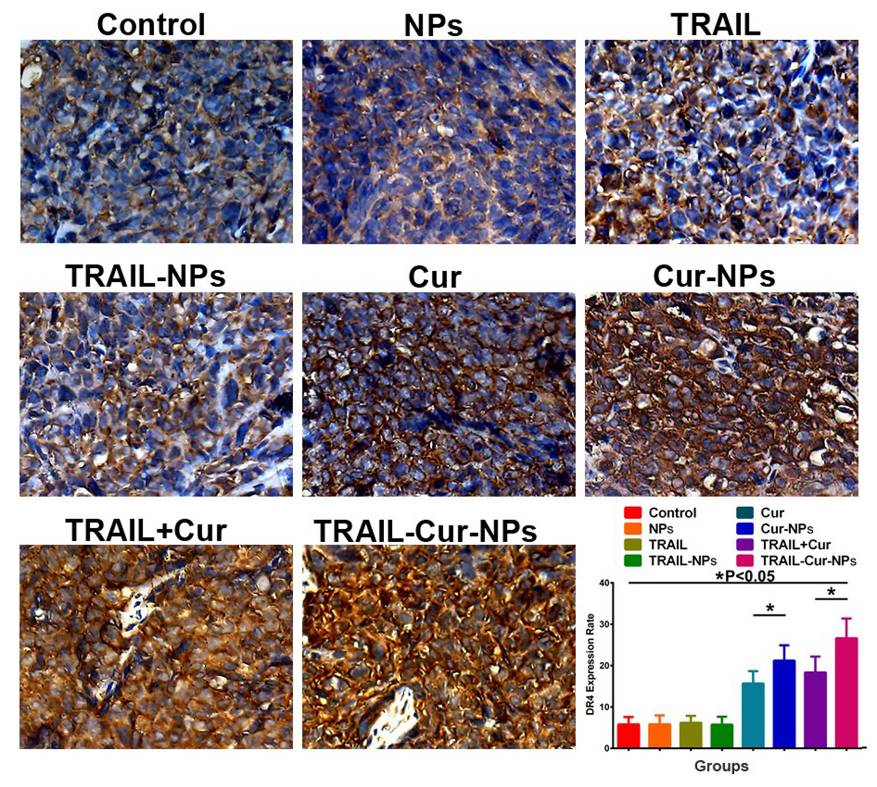
**

**Figure S6.** DR4 immunohistochemically staining of HCT116 tumors in different treatment groups.

**
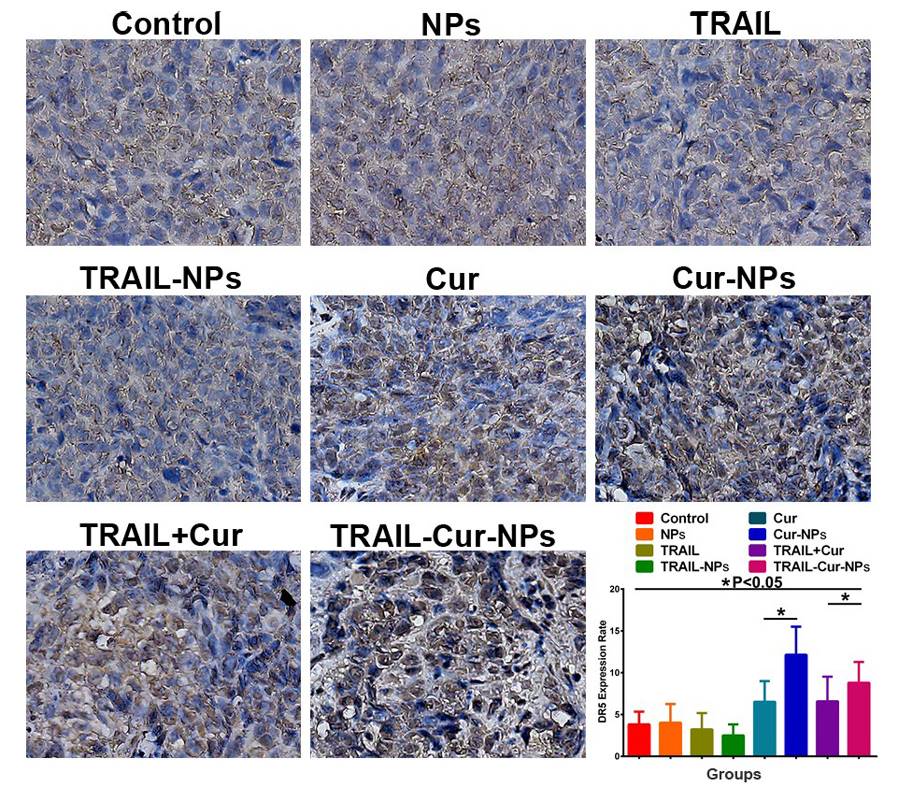
**

**Figure S7.** DR5 immunohistochemically staining of HCT116 tumors in different treatment groups.

**
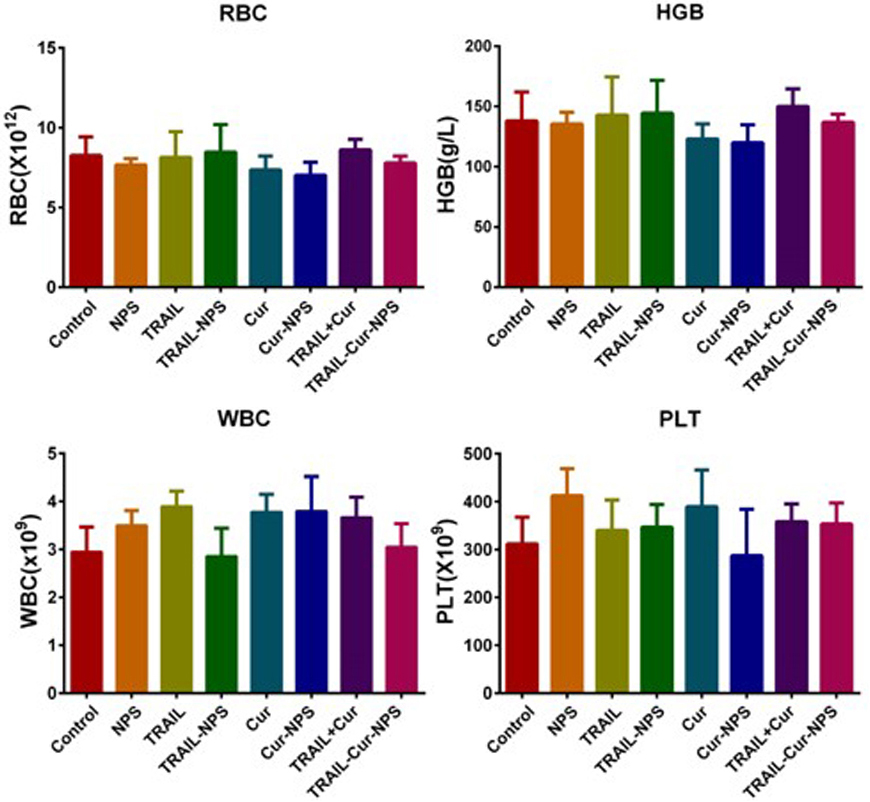
**

**Figure S8.** Complete blood count in different treatment groups.


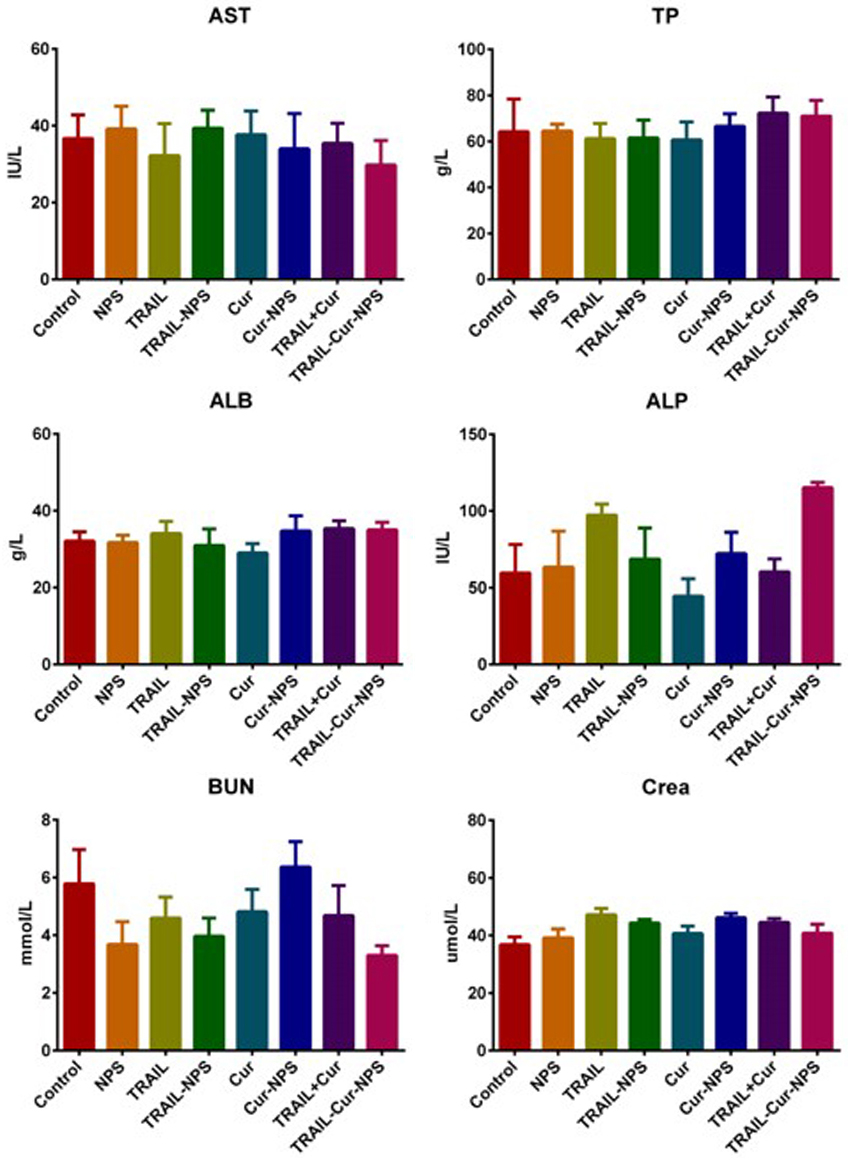


**Figure S9.** Serum chemistry profile and kidney function in different treatment groups.

**
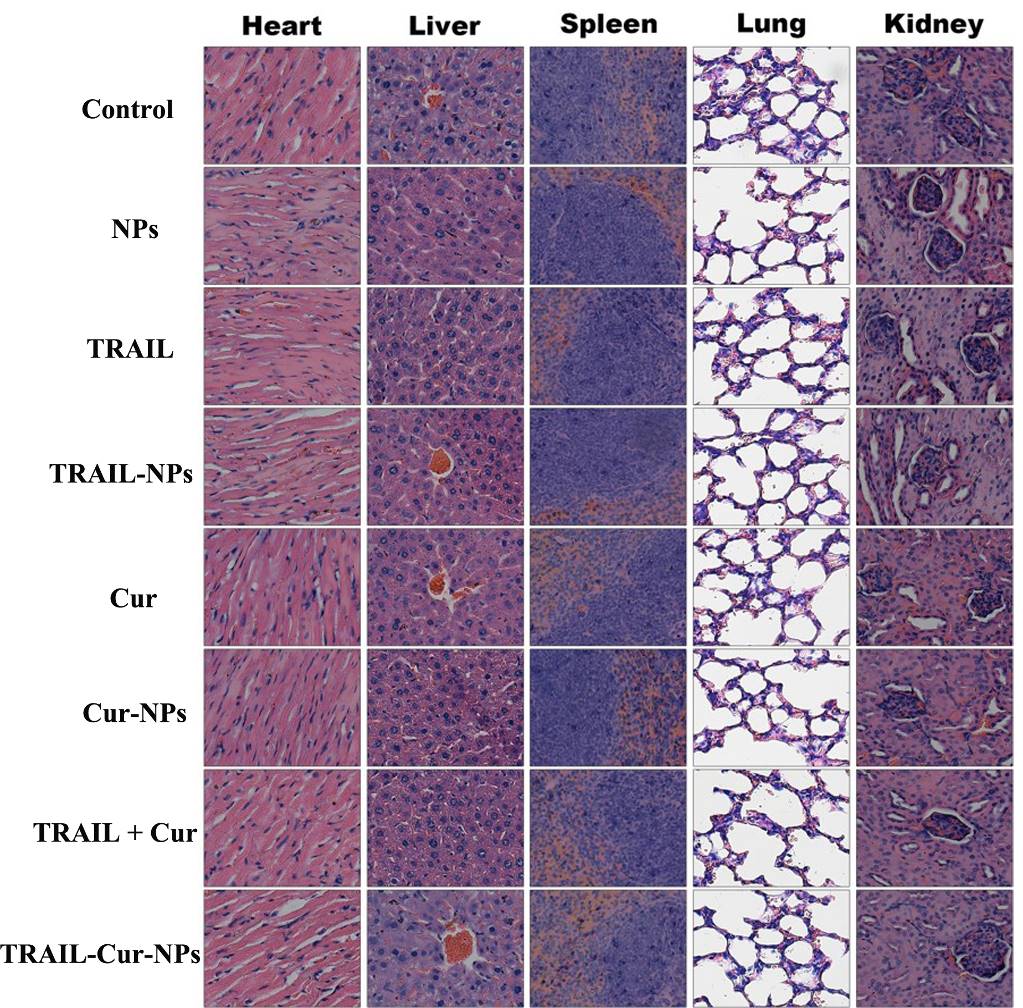
**

**Figure S10.** H&E staining of major organs (heart, liver, spleen, lungs, and kidneys) in different treatment groups.
